# Barriers to establishing teledermatoscopy in primary health care in Sweden

**DOI:** 10.1186/s12875-024-02678-w

**Published:** 2024-12-17

**Authors:** Nils Hernström, Åsa Ingvar

**Affiliations:** 1 Dermatology and Venereology, Department of clinical sciences, Lund University Skin Cancer research group (LuScaR), Lund University, Skåne University Hospital, Raffinadgatan 4, Lund, 222 35 Sweden; 2 Måsen Primary Health Care Center, Lund, Sweden

**Keywords:** Teledermatology, Teledermatoscopy, Primary care, Implementation

## Abstract

**Introduction:**

Teledermatoscopy (TDS) has proven to be effective and reliable for diagnosis of skin malignancies. The factors that determine the success of implementation of TDS are largely unknown.

**Objectives:**

To investigate barriers to implementation of TDS in primary health care (PHC) at center and individual level.

**Methods:**

Following introduction of TDS, cross-sectional quantitative data and free text comments were collected by surveys sent to PHC centers and PCH practitioners. Successful implementation was defined as regularly sent cases at center level and self-reported usage at individual level. Factors associated with implementation were evaluated with Chi-square, Kruskal-Wallis test and logistic regression.

**Results:**

93/117 (78.2%) of PHC centers and 239/725 (32.9%) of PHC practitioners answered the surveys. 54.8% (*n* = 51) of PHC centers and 64.3% (*n* = 153) of PHC practitioners had implemented TDS.

There was a strong association between hardware arrival before introduction and TDS usage at center level (OR 6.0; 95% CI 1.5–24.3). At individual level, male sex was positively associated with usage (OR 1.9; 95%, CI 1.0–3.4), and for every year of increased age, the chance of using TDS decreased with 3% (OR 1.0, 95% CI 0.9–1.0). No other factor was associated with implementation. “Good” was the most common overall impression (54.8%), and the majority found no problems using the system (> 85%). The most common complaint was technical issues followed by no added value.

**Conclusions:**

Successful implementation of TDS was strongly associated with hardware arrival at center level, and to male sex and younger age at individual level. Satisfaction was overall high.

**Supplementary Information:**

The online version contains supplementary material available at 10.1186/s12875-024-02678-w.

## Background

Starting in the spring of 2022, continuing for approximately one year, the southernmost health care region in Sweden, *Region Skåne*, implemented a teledermatology/teledermatoscopy (TDS) system in all its primary healthcare (PHC) centers ( *n* = 167) [1]. TDS is a digital consultation system in which referrals containing information and images of suspicious skin lesions are sent from primary healthcare providers to dermatology departments. This study evaluates the implementation to learn what barriers and problems exist for successful implementation and well-functioning organization and use of TDS.

TDS implementation commenced following a national recommendation to address the increasing burden of skin cancer and to improve skin lesion diagnostics. Skin cancer incidence, including the most aggressive type of skin cancer, melanoma (MM), has more than tripled in Sweden since 1990 and is continuously increasing in the older population but has begun to stabilize and even decline in the younger population since about 10 yearsL [2–4]. Alarmingly, Sweden has one of the world’s highest incidences of MM, which is now the 5th most common malignant tumor type in the population [2]. Of special concern, MM causes almost 90% of all skin cancer related deaths and is one of the most common cancers in the younger population [2, 5]. Early diagnosis is essential to maintain a favorable prognosis and possibility to curative treatment [6–9]. Unfortunately, diagnosing MMs can be a challenge, especially in early stages and in PHC. This results in a low diagnostic accuracy, with risk of both overlooked MM and a high number of benign lesions unnecessarily excised [10].

TDS has been shown to improve medical triage, decrease time to surgery and decrease skin cancer treatment costs [11–14]. For skin malignancies, TDS has been shown to be a reliable instrument for diagnosis with a good (but not perfect) agreement with face-to-face examinations by dermatologists [11, 15, 16]. A Swedish study found TDS referral to be as efficient as traditional referral [17] .In a systematic review by Dovigi et al. in 2020, it was found that implementation of TDS was mainly obstructed by technical difficulties, time consumption, and low integration with existing workflows and health records, whereas facilitation was driven by its effectiveness, convenience, and ease of use [18]. Satisfaction with TDS has been studied previously among dermatologists and patients, but rarely among PHC practitioners [18–21]. Some smaller studies that did evaluate primary care practitioners’ satisfaction with TDS found it to be high but provided no deeper analysis of the underlying determinants [22–24]. With this study we aim to increase knowledge on how to implement and organize TDS successfully by investigating which factors impaired the implementation and use of TDS on individual PHC practitioner and PHC center level in Region Skåne.

The TDS system implemented in Region Skåne is a so-called store-and-forward system, with referrals from primary care being stored for later evaluation by dermatologists. The TDS-system consists of hardware: mobile phones and attachable dermatoscopes, and software: a mobile application and a web-based platform [23]. The system is primarily intended for examination of pigmented suspected malignant lesions. TDS has been organized in Region Skåne by instituting an operations manager, a systems manager, and account administrators. The operations manager overviews the full system, monitors that referrals are assessed, performs quality control, and receives feedback and incidental reports. The system manager overviews the technical aspects, including alignment with regulations, and effectuates system developments. There is one account administrator in every PHC center and dermatology department overviewing the TDS locally.

The implementation of TDS consisted of distribution of hardware and software to all PHC centers, and education of assessors, account administrators and PHC users. The introduction for users consisted of a 4-hour session with presentation of the system, intended use, benefits and risks with TDS, and recommended examination of patients referred by TDS. The focus was on the two perceived major weaknesses of using TDS; correct imaging of lesions and recommended procedures to uphold patient safety.

## Methods

To understand the determinants of successful TDS implementation, we performed a survey-based retrospective cross-sectional study. A questionnaire was created specifically for this study and managed using the Research electronic data capture (REDCap) tool hosted at Lund University [25, 26].

**Fig. 1:**
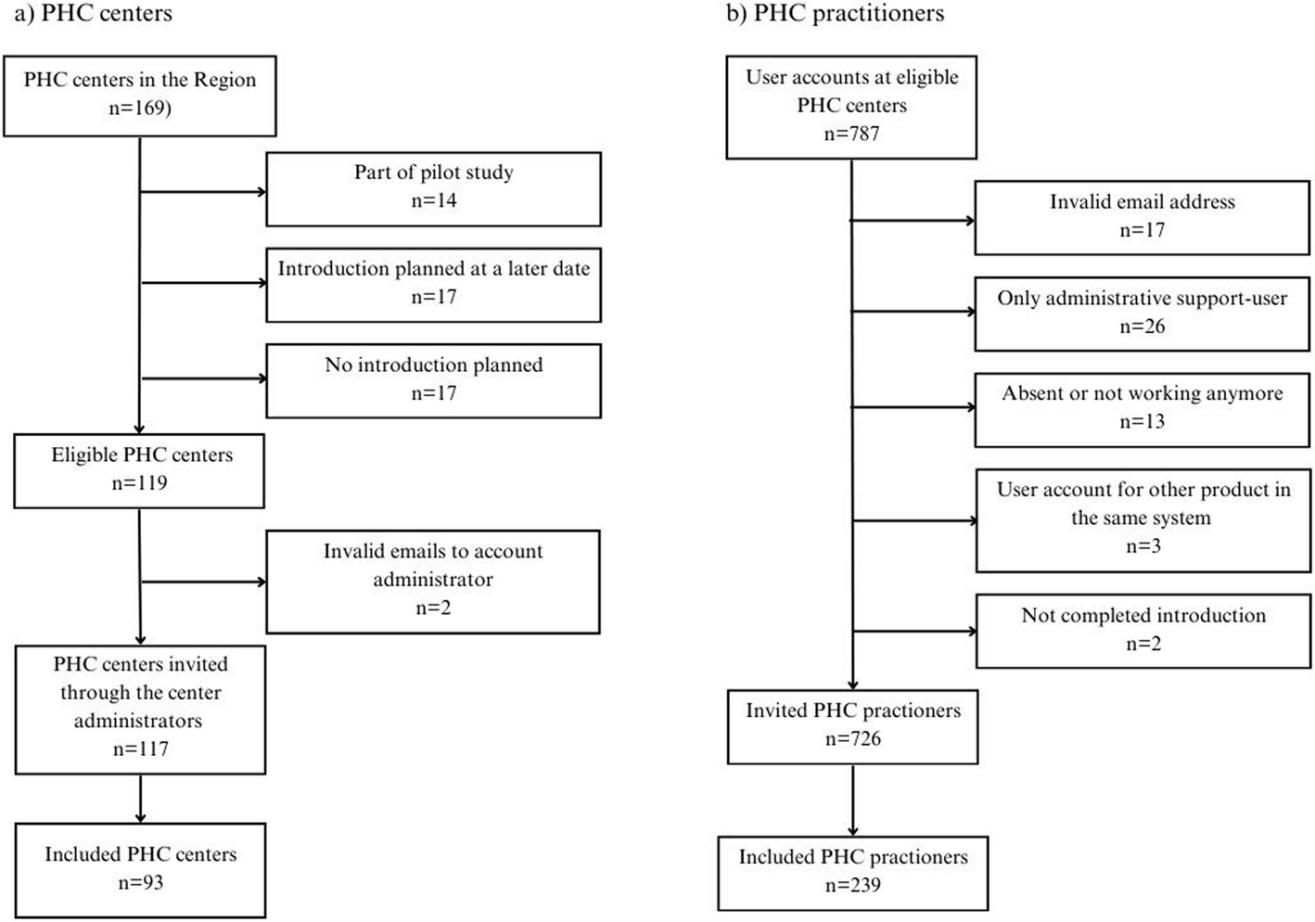
Title: Flowchart of the inclusion process Legend: PHC = Primary health care

The survey was distributed to all PHC users (*n* = 787) (supplement 1) and all account administrators (*n* = 119) (supplement 2) belonging to a PHC center introduced to TDS during 2022 in Region Skåne. PHC centers and affiliated users were excluded if they had been introduced before 2022 during a pilot study in autumn 2020 (*n* = 14), completed their introduction in the beginning of 2023 (*n* = 17) or had no introduction yet (*n* = 17) (Fig. 1).

Two different surveys were distributed at a minimum of 3 months after completed introduction. One directed to PHC center administrators and one to all registered users. All eligible participants were sent the survey and a reminder after 2 and 4 weeks. The center administrator survey consisted of general questions such as number of patients listed at the PHC center, number of physicians attending the TDS introduction, if hardware had arrived and was functioning before the introduction, and if a skin check clinic had been established at the PHC. PHC practitioners answered questions without identification data and specific to being an individual user, such as age, sex, professional title, previous training in dermatoscopy, if they used TDS and to which extent, and impression of the hardware and software.

### Statistical methods

Data was analyzed using StataCorp. 2021. Stata Statistical Software: Release 17. College Station, TX: StataCorp LLC. For the purpose of analyses, we considered the implementation of TDS at a PHC center successful if the center sent one or more TDS case(s) per calendar month in any 3 of the first 5 months following introduction. On an individual level, an active user was determined by the PHC practitioners self-reported usage of the system. Descriptive statistics were compiled and compared active to inactive PHC center or PHC practitioners using Pearson’s chi^2^ tests (categorical data) or Kruskal-Wallis tests (continuous data). A *p*-value < 0.05 was considered statistically significant. Odds ratios (OR) and 95% confidence intervals (CI) were estimated using univariate and multivariate logistic regression to assess the association between exposure variables and being an active PHC center or PHC practitioner. Answers to open questions were grouped into appropriate themes and presented using general descriptions and specific examples.

### Ethics

The study was approved by the Swedish Ethics review authority, nr 2020–04763. The participation was fully voluntary, and the survey responses were collected without identification data. However, by using a combination of data collected about each respondent, it would be theoretically possible to identify a PCP. Therefore, the data was kept in a secure data storage platform which only the relevant researchers could reach. The data was also not of sensitive character and would presumably not have a significant impact upon a person if they were identified.

## Results

A total of 119 eligible PHC centers were identified, but 2 PHC centers had not provided valid emails to account administrators. Hence, 117 PHC center surveys were sent and 93 (78.2%) were returned (Fig. 1). Of the responding centers, 54.8% (*n* = 51) had established TDS (Table 1). 92.2% of PHC centers with successful implementation of TDS reported that hardware had arrived and was functioning at the time of the introduction, compared to 73.8% for PHC centers that did not implement TDS (*p* = 0.05). A greater proportion of PHC centers with successful implementation of TDS also had an established skin check clinic (47.1% vs. 31%, *p* = 0.11) and were of large size (41.2% vs. 23.8%, *p* = 0.18).

**Table 1:** Characteristics of primary health care centres with and without successful implementation^a^ of teledermatoscopy

	Successful implementation of TDS at PHC centres				
	No		Yes		
	Number	Percent	Number	Percent	*p*-value
Total	42	45.2%	51	54.8%	
Skin check clinic established					*p* = 0.11^P^
No	29	69.0%	27	52.9%	
Yes	13	31.0%	24	47.1%	
Appointed person responsible for TDS present at PHC center					*p* = 0.42^P^
No	3	7.1%	2	3.9%	
Yes	38	90.5%	45	88.2%	
Don’t know	1	2.4%	4	7.8%	
PHC center size					*p* = 0.18^P^
Small (2000–7000 patients)	15	35.7%	16	31.4%	
Medium (7500–9900 patients)	17	40.5%	14	27.5%	
Large (10000–25000 patients)	10	23.8%	21	41.2%	
Number of doctors introduced					*p* = 0.76^P^
1–2	18	42.9%	18	35.3%	
3–4	11	26.2%	19	37.3%	
>4	11	26.2%	12	23.5%	
Don’t know	2	4.8%	2	3.9%	
Hardware received and functioning at time of introduction					*p* = 0.05^P^*
No	10	23.8%	4	7.8%	
Yes	31	73.8%	47	92.2%	
Don’t know	1	2.4%	0	0.0%	

*PHC* primary health care

*TDS* teledermoscopy

*SD* Standard deviation

*P* Pearson chi square-test

*K* Kruskal-Wallis test

PHC center size = Tertiles of PHC centers by number of listed patients

*Statistically significant difference between the groups

^a^Successful implementation = at least 1 case sent via TDS system for at least 3 of the 5 first calendar months following introduction

The PHC practitioner survey was distributed to all identified 787 user accounts. 61 users were excluded due to either of the following: 26 were mere administrative support-users, 17 had invalid email addresses, 13 were absent or had changed jobs, 3 used the system for another product, and 2 had not completed introduction. Consequently, 726 eligible PHC practitioners were invited and 239 (32.9%) answered the survey and were included in the analyses (Fig. 1). 64.3% (*n* = 153) of PHC practitioners used TDS and 35.6% (*n* = 85) did not (Table 2). The mean age was 43.7 years for TDS users and 46.6 years for non-users. A slightly greater proportion of users were men (39.9% vs. 27.1%).

**Table 2:** Descriptive characteristics of primary health care practitioners who used teledermatoscopy after introduction and those who did not

	Use of TDS						
	No		Yes				
	Number	Percent	Number			Percent	
Total	85	35.7%	153			64.3%	
Age, years. Mean (SD)	46.6	(10.7)	43.7			(9.0)	*p* = 0.03^T^*
Sex							*p* = 0.11^P^
Female	61	71.8%	89			58.2%	
Male	23	27.1%	61			39.9%	
Don’t want to specify	1	1.2%	3			2.0%	
Professional title							p = < 0.005^P^*
PHC practitioner (consultant)	45	52.9%	107			69.9%	
Resident physician	20	23.5%	35			22.9%	
Employed by the hour	0	0.0%	4			2.6%	
Intern	1	1.2%	5			3.3%	
Other^a^	19	22.4%	2			1.3%	
Dermatoscopy training							*p* = 0.30^P^
No	49	57.6%	81			52.9%	
Yes	35	41.2%	72			47.1%	
Don’t know	1	1.2%	0			0.0%	

*PHC* primary health care

*TDS* teledermoscopy

*T* Two-sample t-test

*P* Pearson chi square-test

*Statistically significant difference between the groups

a = 1 missing data

Results of logistic regression analyses at individual and center level are presented in Table 3. At PHC center level, there was a strong association between “hardware arrived and functioning at time of introduction” and a successful implementation of TDS (OR 5.9; 95% CI 1.5–24.3). The other factors were not associated with successful TDS implementation at center level. At individual level, the multivariate OR for becoming a TDS user after introduction was almost double for males compared to females (OR 1.9; 95% CI 1.0–3.4) and significantly decreased by 3% (OR 1.0, 95% CI 0.9–1.0) for every year of increased age of the PHC practitioner.

**Table 3:** Univariate and multivariate logistic regression analyses estimating association between the analysed factors and the probability of using teledermatoscopy in primary care on (a) primary health care center level, (b) individual level

	Univariate	Multivariate
	OR (95% CI)	OR (95% CI)
a)	**Primary health care center level**	
Skin check clinic established		
No	1	1
Yes	2.0 (0.8–4.7)	2.5 (0.9–6.7)
PHC center size		
Small	1	1
Medium	0.8 (0.3–2.1)	0.5 (0.2–1.5)
Large	2.0 (0.7–5.5)	1.7 (0.5–5.9)
Number of doctors introduced		
1–2	1	1
3–4	1.7 (0.6–4.6)	2.4 (0.8–7.8)
>4	1.1 (0.4–3.1)	1.1 (0.3–3.8)
Hardware received and functioning at time of introduction		
No	1	1
Yes	3.8 (1.1–13.2)^a^	6.0 (1.5–24.3)^a^
Appointed person responsible for TDS present at PHC center	1.8 (0.3–11.2)	N/A
b)	**Individual level**	
Sex		
Woman	1	1
Man	1.8 (1.0–3.2) ^a^	1.9 (1.0–3.4)^a^
Don’t want to specify	2.1 (0.2–20.2)	2.1 (0.2–21.6)
Age (years)	1.0 (0.9–1.0)	1.0 (0.9–1.0)^a^

*PHC* primary health care

*TDS* teledermoscopy

*OR* Odds Ratio, *CI* Confidence interval

^a^Statistically significant association between exposure and chance of using TDS

**Fig. 2:**
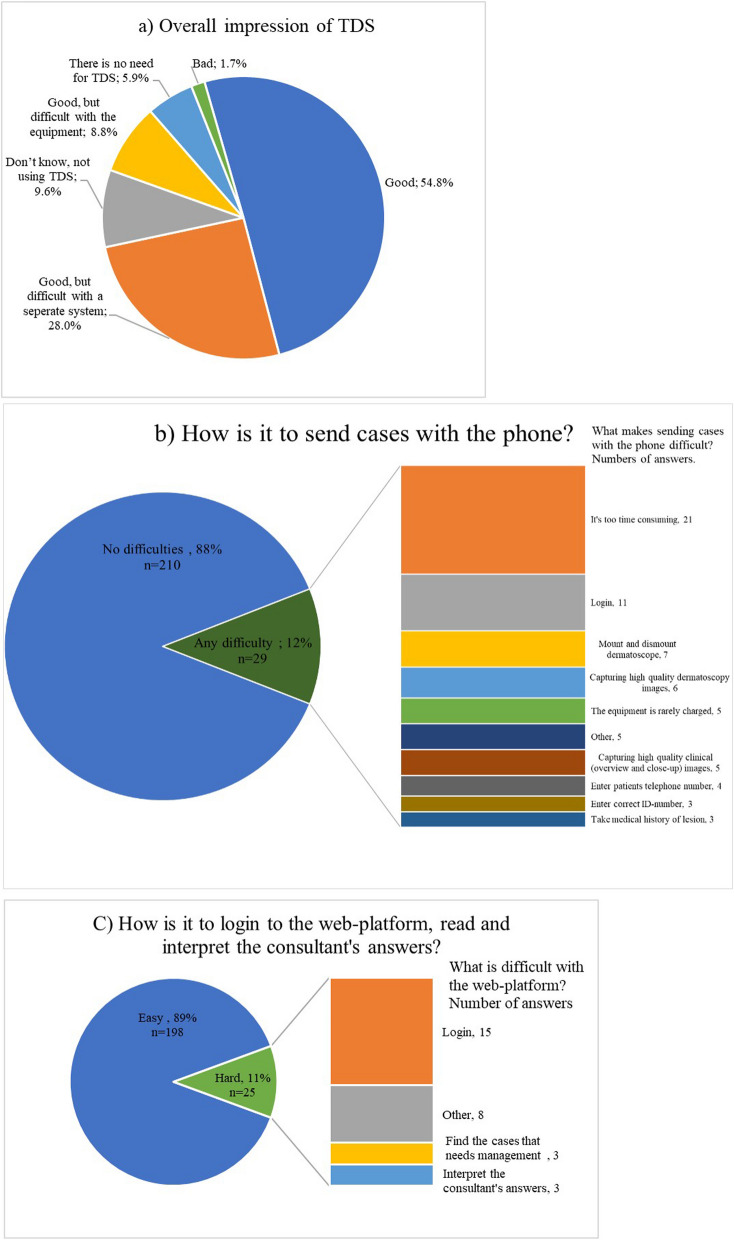
Pie charts of Primary health care center (PHC) practitioners’ impression of teledermatoscopy (TDS) Legend: **a** Overall impression of TDS. TDS = teledermatoscopy (**b**) Primary health care center (PHC) practitioners’ impression of phone, attachable dermatoscope and mobile application. To the right specification if stated any difficulty, multiple choices possible (**c**) Primary health care center (PHC) practitioners’ impression of web-platform To the right specification if stated hard, multiple choices possible

The general impression of using TDS and the TDS system is summarized in Fig. 2a. The most common overall impression was “Good” (54.8%) followed by “Good, but difficult to work in a separate system” (28.0%). The vast majority found no problems with using the phone/app/dermatoscope (87.9%) or the web platform (88.8%) (Fig. 2b and c). Of those who found the phone difficult to use (12.1%), 72.4% found it “Too time consuming”, 37.9% had issues with “Login” (*n* = 11) and/ or “Capturing high quality clinical/dermatoscopic images” (*n* = 11) (Fig. 2b). Regarding users who found it difficult working with the web-based platform (11.2%), the majority found the “Login” (60%) difficult (Fig. 2c).

Ninety six free-text comments were grouped into 7 themes as shown in Table 4. Most comments, 20.8% (*n* = 20) concerned “Technical issues” like: “Have had trouble obtaining focused images.”, “TDS is time consuming and you feel unprofessional when you fumble with mounting and dismounting the dermatoscope”. About equal number of PHC practitioners submitted comments either in the “Good” theme or the “Unnecessary, no added value” theme. The latter exemplified by: “Provides no added value for me, feels like dermatology departments only transfers work to primary care”. The fourth most common theme was organizational and system integration issues (14.3%, *n* = 13). These comments related either to the hurdles caused by the non-existing integration of the TDS system with the electronic medical records or a wish for automating referrals for face-to-face examination to dermatologist if recommended in the TDS consultation report.

**Table 4:** Categorization of free text comments

Category of free text comment	Number	Percent
Technical issues	20	20.8%
Unnecessary, no added value	14	14.6%
Organizational and system integration issues	13	13.5%
Lack of resources / takes too much resources	9	9.4%
Not applicable to all skin conditions	6	6.3%
Implementation/introduction	5	5.2%
Good	15	15.6%
No comments / No usage	14	14.6%

## Discussion

To investigate the hurdles of implementing TDS in a health care system on a center and an individual level, we performed a survey-based retrospective cross-sectional study. We found that about 55% of PHC centers and about 64% of individual users implemented TDS after introduction, according to our definition. Having received the TDS hardware and software before the introduction was most strongly associated with successfully implementing TDS on a PHC center level. On an individual level, male sex and younger age both increased the probability to use TDS. Overall, the majority of respondents had a good overall impression of TDS, although about 12% had technical issues, and/or found it time-consuming and/or troublesome to manage TDS in a system that is not integrated with the electronic medical record.

This study shows that PHC centers were almost 6 times more likely to have a successful implementation of TDS if they had received the TDS hardware and downloaded the TDS app before the introduction. This is in line with results from the review by Dogvini et al. where 30% of the studies reported barriers within the organizational structure and the study by Orruno et al. that found that infrastructure significantly influenced the intention to use TDS [18, 27]. Consequently, making sure PHC centers have the prerequisites to start using TDS directly after introduction seems to be fundamental for a successful implementation. However, there is a possibility that well organized PHC centers with a positive attitude to TDS confounded this association since they might have been more successful in acquiring the materials before the introduction.

Neither PHC center size nor the number of introduced PHC practitioners were significantly associated with successful implementation of TDS. Furthermore, there was no statistically significant difference between PHC centers with or without an established skin check clinic, although this was more common in PHC centers that were successful in implementing TDS (47.1% vs. 31.0%, *p* = 0.11). We were unable to find studies examining these aspects of implementation of teledermatology or other similar digital technologies in PHC. These results might be impacted by our definition of successful implementation that, if it is too broadly defined, might cause non-differential misclassification and dilution of results.

It was found in this study that males were almost twice more likely to use TDS and that for every one year of increased age, the likelihood of using TDS decreased by 3%. This translates to a 30% decreased probability of using TDS for a 50-year-old PHC practitioner compared to a 40-year-old PHC practitioner. Sex and age have not been shown to be associated with successful implementation of TDS before [18]. In articles investigating implementation of other eHealth technologies (e.g., electronic health records and telemedicine) there have been contradictory results regarding the association between technology adoption and age and sex [28–31]. Jacobs et al. acknowledged the unclear association to sex and age but also found that younger clinicians had a more positive attitude to new technologies and that increasing years of professional experience could negatively impact adoption [29].

Amongst the respondents, 22 persons stated “other” as professional title. In this group, a much bigger proportion (90.4%) compared to the total stated no use of TDS. We suspect that despite detailed instructions, some non-physicians replied to the survey, making association between use of TDS and professional title difficult to interpret.

The absolute majority of users of the TDS system (> 90%) found the TDS system to be “Good”. Only 7.6% (*n* = 18) found the implementation of TDS “Bad” or “Unnecessary/ no added value”. This is in line with the earlier studies of TDS satisfaction and specifically studies assessing satisfaction with TDS among GPs in Denmark and Belgium [22, 23]. Furthermore, most users were handling the phone and web-platform with ease, but a significant proportion had difficulties, mainly technical problems also reflected in the free text comments. We did not study if this impacted the usage of TDS, but earlier studies suggest that technical problems and low perceived ease of use might be a significant barrier [18, 27]. The 13 comments in the theme “organizational and system integration issues” indicates that the current organization and use of a separate system for TDS, with no integration to the electronic medical records, is not optimal. Healthcare organizational hurdles in Region Skåne prohibit a more automated process for forwarding TDS referrals for face-to-face examinations to dermatology units when this is recommended. This results in an increased administrative burden on the PHC practitioner. These findings are consistent with findings in the review of facilitators and barriers to TDS implementation [18].

No complaints were received either at individual or center level about liability or reimbursement problems, which is contradictious to earlier studies [18, 28]. Possibly this is due to the structure of the Swedish health care system and the financing of this project.

## Limitations

Most importantly, only 32.9% of invited users in the TDS system responded to our survey despite 2 reminders. Consequently, there might be a selection bias in the responses we received. First of all, we do not know how many of non-responders that were also non-users. One might speculate that there is a larger proportion of non-users of TDS among the non-responders and, if so, the uptake of TDS among individual users would be even lower than what was found in this study. Furthermore, being motivated to respond to the questionnaire might hypothetically be driven by either being very satisfied or very dissatisfied with the system. Unfortunately, we cannot with certainty know if this selection bias is present or in which direction it distorts our results. Furthermore, there is no accepted definition for successful implementation on center level and our definition (at least one TDS consultation/ month for at least three out of five months following the introduction) might be too inclusive. However, a more stringent definition, we argue, might result in incorrectly categorizing many small PHC centers as inactive.

## Conclusions

The adoption of TDS after introduction is about 55% at center level and 60% at an individual level. It is important to understand factors affecting the willingness to use TDS. We found that hardware and software arrival before introduction impacted implementation on PHC center most and that younger age and male sex increased the chance of being a TDS user. Users of TDS were generally satisfied with TDS but some experienced technical issues (most often Login) and insufficient integration to medical records and workflow. These factors should be taken into consideration when introducing TDS, or similar digital e-health systems/ devices, to a health care system.

## Supplementary Information

Supplementary Material 1.

Supplementary Material 2.

## Data Availability

The datasets used and/or analyzed during the current study are available from the corresponding author on reasonable request.

## Abbreviations

CI: Confidence Intervals
MM: Melanoma
OR: Odds Ratios
PHC: Primary Healthcare
TDS: Teledermatology/Teledermatoscopy
