# Supplementary material for: Barriers to establishing teledermatoscopy in primary health care in Sweden
Source: BMC Prim Care. 2024 Dec 17;25:417. doi: 10.1186/s12875-024-02678-w (PMC11653750; doi:10.1186/s12875-024-02678-w)
Supplement: Supplementary file 1 — Supplementary Material 1. [file 12875_2024_2678_MOESM1_ESM.pdf]

Supplement file 1. Survey for Primary Health Care Practitioners

Translated from Swedish to English.

#	Variable / Field Name	Field Label<br>*Field Note*	Field Attributes (Field Type, Validation, Choices, Calculations, etc.)
Instrument: Läkare (lkare)		☐ Enabled as survey	
1	[record_id]	Record ID	text
2	[vc]	Which health care center do you work at?	text, Required
3	[sex_dr]	Are you a man or a woman?	radio, Required<br>0 Woman<br>1 Man<br>2 I don´t want to specify
4	[age_dr]	How old are you?	text (number, Min: 23, Max: 80), Required
5	[dr_typ]	What's your professional title?	radio, Required<br>0 Intern<br>1 Resident<br>2 PHC practitioner (consultant)<br>3 Employed by the hour<br>4 Other
6	[utb_dermatoskopi]	Except the introductio to teledermatoscopy, have you completed any other course of dermatoscopy?	radio, Required<br>0 No<br>1 Yes<br>2 Don't know
7	[going]	Have you sent any patient cases (in addition to the test case) via teledermatoscopy?	radio, Required<br>0 No<br>1 Yes
8	[regelbunden]<br>Show the field ONLY if: [going] = '1'	Do you regularly (at least one case every two months) send patient cases via teledermatoscopy?	radio, Required<br>0 No<br>1 Yes<br>2 Periodically
9	[phone]	How is it to send cases with the phone?	radio, Required<br>0 Easy<br>1 Hard
10	[phone_diffic]<br>Show the field ONLY if: [phone] = '1'	What makes sending cases with the phone difficult?	checkbox, Required<br>0 phone_diffc0 Login<br>1 phone_diffc1 Enter correct ID number<br>2 phone_diffc2 Enter patients telephone number<br>3 phone_diffc3 Take medical history of lesion<br>4 phone_diffc4 Capturing high quality clinical (overview and close-up) images<br>5 phone_diffc5 Capturing high quality dermatoscopy images<br>6 phone_diffc6 Mount and dismount dermatoscope

			7 phone_diffc7 The equipment is rarely charged<br>8 phone_diffc8 It's too time consuming<br>9 phone_diffc9 Other
11	[telephone_other]<br>Show the field ONLY if: [phone_diffc(9)] = '1'	If you have stated ”other” on difficulties with sending cases with the phone, please specify here what you meant	notes
12	[web]	How is it to login to the web-platform, read and interpret the consultants answer?	radio<br>0 Hard<br>1 Easy
13	[web_diffic]<br>Show the field ONLY if: [web] = '0'	What is difficult with the web-platform?	checkbox<br>0 web_diffc0 Login<br>1 web_diffc1 Find the cases that needs managment<br>2 web_diffc2 Interpret the consultant's answer<br>3 web_diffc3 Other
14	[annat_web_diffic]<br>Show the field ONLY if: [web_diffc(3)] = '1'	If you have stated ”other” on difficulties with the web-platform, please specify here what you meant<br>.	notes
15	[svar_bevak]	How do you monitor answers to cases in Dermicus?	checkbox, Required<br>0 svar_bevak 0 Notification by mail<br>1 svar_bevak 1 Secretary monitors the answers and sends to me<br>2 svar_bevak 2 In my schedule<br>3 svar_bevak 3 Next skin check clinic<br>4 svar_bevak 4 Other
16	[fritext_bevaka]<br>Show the field ONLY if: [svar_bevak(4)] = '1'	If you stated "other" on how you monitor answers in Dermicus, please indicate how you monitor answers here.	text
17	[overarch_tele]	How is your overall impression of teledermatoscopy	checkbox, Required<br>0 overarch_tele 0 Good<br>1 overarch_tele 1 Good, but difficult with the equipment<br>2 overarch_tele 2 Good, but difficult with a separate system<br>3 overarch_tele 3 Bad<br>4 overarch_tele 4 There is no need for teledermatoscopy<br>5 overarch_tele 5 Don't know, not using teledermatoscopy
18	[fritext_bad]<br>Show the field ONLY if: [overarch_tele(3)] = '1'	What do you think is bad about teledermatoscopy and/or the teledermatoscopy system?	text
19	[fritext]	Do you have other opinions on how the teledermatoscopy system works? Possibility to comment in free text on the system as it looks today or if there is something you are missing.	notes

20	[lkare_complete]	Section Header: *Form Status*<br>Complete?	dropdown<br>0 Incomplete<br>1 Unverified<br>2 Complete
