# Supplementary material for: Barriers to establishing teledermatoscopy in primary health care in Sweden
Source: BMC Prim Care. 2024 Dec 17;25:417. doi: 10.1186/s12875-024-02678-w (PMC11653750; doi:10.1186/s12875-024-02678-w)
Supplement: Supplementary file 2 — Supplementary Material 2. [file 12875_2024_2678_MOESM2_ESM.pdf]

Supplement file 2. Survey for Primary Health Care Centers

Translated from Swedish to English.

<table>
<tr><th>#</th><th>Variable / Field Name</th><th>Field Label
Field Note</th><th>Field Attributes (Field Type, Validation, Choices, Calculations, etc.)</th></tr>
<tr><td colspan="4">Instrument: Kontoadministratör (kontoadministratr) Enabled as survey</td></tr>
<tr><td>1</td><td>[record_id]</td><td>Record ID</td><td>text</td></tr>
<tr><td>2</td><td>[vc_namn]</td><td>What is the name of your health care center?</td><td>text, Required, Identifier</td></tr>
<tr><td>3</td><td>[listning]</td><td>How many listed patients do you have at your health center (rounded to the nearest 100)?</td><td>text (number, Min: 2000, Max: 30000), Required</td></tr>
<tr><td>4</td><td>[intro_month]</td><td>In which (or which) months did your health center receive an introduction to teledermatoscopy?</td><td>checkbox, Required<table><tr><td>0</td><td>intro_month 0</td><td>April</td></tr><tr><td>1</td><td>intro_month 1</td><td>August</td></tr><tr><td>2</td><td>intro_month 2</td><td>September</td></tr><tr><td>3</td><td>intro_month 3</td><td>October</td></tr><tr><td>4</td><td>intro_month 4</td><td>November</td></tr><tr><td>5</td><td>intro_month 5</td><td>February 2023</td></tr><tr><td>6</td><td>intro_month 6</td><td>Don’t know</td></tr></table></td></tr>
<tr><td>5</td><td>[nr_dr]</td><td>How many doctors completed the full introduction (the regional cancer center online course + introduction course)?</td><td>radio, Required<table><tr><td>0</td><td>0</td></tr><tr><td>1</td><td>1</td></tr><tr><td>2</td><td>2</td></tr><tr><td>3</td><td>3</td></tr><tr><td>4</td><td>4</td></tr><tr><td>5</td><td>>4</td></tr><tr><td>6</td><td>Don’t know</td></tr></table></td></tr>
<tr><td>6</td><td>[prickmottagning]</td><td>Do you have a "skin check clinic" at your health care centre? Specific clinic time for assessment of suspected skin lesions.</td><td>radio, Required<table><tr><td>0</td><td>No</td></tr><tr><td>1</td><td>Yes, since before we started using teledermatoscopy</td></tr><tr><td>2</td><td>Yes, we started a skin check clinic as we started using teledermatoscopy</td></tr><tr><td>3</td><td>No, but we are planning to start a skin check clinic</td></tr></table></td></tr>
<tr><td>7</td><td>[material]</td><td>Had you received phone, mobile dermatoscope and the application before the time of introduction?</td><td>radio, Required<table><tr><td>0</td><td>No</td></tr><tr><td>1</td><td>Yes</td></tr><tr><td>2</td><td>Don’t know</td></tr></table></td></tr>
<tr><td>8</td><td>[ansvar_per]</td><td>Do you have an appointed person responsible for TDS present at your PHC center ?
Someone who makes sure that the phone and dermatoscope are charged and that immersion liquid is ordered</td><td>radio, Required<table><tr><td>0</td><td>No</td></tr><tr><td>1</td><td>Yes</td></tr><tr><td>2</td><td>Don’t know</td></tr></table></td></tr>
<tr><td>9</td><td>[fritext]</td><td>Is there anything else that has hindered you from getting started with teledermatoscopy?</td><td>notes</td></tr>
<tr><td>10</td><td>[kontoadministratr_complet e]</td><td>Section Header: *Form Status*
Complete?</td><td>dropdown<table><tr><td>0</td><td>Incomplete</td></tr><tr><td>1</td><td>Unverified</td></tr><tr><td>2</td><td>Complete</td></tr></table></td></tr>
</table>
